# Evaluation of Medetomidine-Ketamine for Immobilization of Feral Horses in Romania

**DOI:** 10.3389/fvets.2021.655217

**Published:** 2021-06-21

**Authors:** Ovidiu Roşu, Iulia Melega, Alina L. Evans, Jon M. Arnemo, Susanne Küker

**Affiliations:** ^1^Faculty of Veterinary Medicine, University of Agronomic Sciences and Veterinary Medicine Bucharest, Bucharest, Romania; ^2^Faculty of Veterinary Medicine, University of Agronomic Sciences and Veterinary Medicine Cluj-Napoca, Cluj-Napoca, Romania; ^3^Department of Forestry and Wildlife Management, Inland Norway University of Applied Sciences, Koppang, Norway; ^4^Department of Wildlife, Fish and Environmental Studies, Swedish University of Agricultural Sciences, Umeå, Sweden

**Keywords:** atipamezole, chemical immobilization, field anesthesia, feral horses, ketamine, medetomidine, blood gas analyses

## Abstract

Feral horses are immobilized for a variety of reasons including population control via contraceptives. Although opioid combinations have been successfully used for immobilization of feral horses, there is a need for combinations using drugs that are more readily available and present less of a human health hazard. We evaluated the chemical immobilization with physiological measurements and blood gas analyses of 91 free-ranging feral horses (*Equus ferus caballus*) remotely immobilized with a combination of 30 mg medetomidine and 775 mg ketamine in a single disposable 6 ml dart. During immobilization, heart rate, respiratory rate, rectal temperature, capillary refill time and peripheral oxygen hemoglobin saturation (SpO_2_) were evaluated. In eight horses, arterial blood samples were analyzed to evaluate the blood gases, acid-base status and hematologic variables. Targeted horses presented a wide range of age, size and body condition. Eighty-one horses had an uneventful mean induction of 7.2 min. Eighty-nine horses were immobilized in lateral recumbency with good muscle relaxation and a median recumbency time of 67 min. Ten horses required supplemental ketamine intravenously (x̄ = 434 mg) due to incomplete immobilization. In 58 horses the effects of medetomidine were antagonized with atipamezole intravenously. Increased respiratory rate (>20 breaths/min), increased heart rate (>45 beats/min) and decreased SpO_2_ < 90% were noted in more than half of the individuals, while increased rectal temperature (>39.0°C) was recorded in six animals. Blood parameters showed hypoxemia (<90 mmHg, *n* = 8), hypercapnia (>45 mmHg, *n* = 5), high glucose levels (>134 mmol/L, *n* = 3), increased blood lactate (>1.5 mmol/L), total carbon dioxide, bicarbonate and base excess which further increased in the second sample, whereas SpO_2_ and calcium values decreased. Recoveries were smooth, with one (*n* = 86) or more (*n* = 5) attempts of standing. Eighty-nine recoveries were uneventful, besides one male that showed signs of monoparesis of the left front leg and one mare with signs consistent with exertional myopathy. In conclusion, medetomidine-ketamine provided a reliable immobilization in feral horses over a wide range of body mass and age. However, based on the observed hypoxemia during immobilization, oxygen supplementation is strongly recommended for this protocol.

## Introduction

Management of feral horses (*Equus ferus caballus*) often requires chemical immobilization of individual animals. Although thousands of feral horses have been effectively captured for translocation by physical means (1), chemical capture is considered a more selective, less stressful and safer method (2–4).

The recommended combination for remote drug administration (“darting”) in feral horses is etorphine-xylazine-atropine (5). Etorphine, a highly potent opioid, is classified as a narcotic substance and its use may be strictly regulated or even prohibited in many countries. In addition, etorphine is extremely toxic for humans and carries a high risk for operators. There are only a few reports on non-opioid alternatives for chemical immobilization of feral horses and physiological monitoring was limited to vital signs and pulse oximetry (2–4). Medetomidine-ketamine is likely the most widely used non-opioid protocol for chemical immobilization (5). Medetomidine is an alpha-2 adrenoceptor agonist with sedative and muscle relaxing properties, whereas ketamine is a dissociative anesthetic agent. Due to the potentiating effect of medetomidine, the effective dose of ketamine is reduced. Thus, the total drug volume for remote injection is less than for other ketamine combinations. Additionally, the effects of medetomidine can be counteracted by administration of atipamezole, an alpha-2 adrenoceptor antagonist, in order to reverse the immobilization. In equids, medetomidine-ketamine was used for darting of free-ranging feral horses (4), captive Przewalsk's horses [*Equus ferus przewalskii*; (6, 7)] and free-ranging feral donkeys [*Equus asinus*; (8)]. However, differing approaching methods and settings (helicopter darting vs. captive environment), species differences, low sample sizes (<12) and the use of different doses are making a direct comparison difficult and indicate a lack of available data of medetomidine-ketamine protocols used on feral horses.

In the Danube Delta, Romania, 220 feral horses were chemically immobilized with medetomidine-ketamine between 2013 and 2016 as part of a management plan (2). Preliminary results showed that 30 mg of medetomidine combined with 775 mg of ketamine in a 6 ml dart provided reliable inductions (9 min) and duration of immobilization (70 min) for adult animals of both sexes over a broad range of body mass, age and physiological status. The aim of our present study is to report immobilization data from 91 horses which have been successfully darted once with a fixed dose of medetomidine-ketamine and blood gas, acid-base and hematological values.

## Materials and Methods

### Animals and Study Area

The present study was approved and carried out in collaboration with the Danube Delta Biosphere Reserve Authority (9) and was part of a population control program, targeting female horses with a Porcine Zona Pellucida immunocontraceptive. The horses inhabited the Grindul Letea area (latitude 45° 20′ N, longitude 29° 30′ E) of Tulcea County, northeast of the Romanian Danube Delta. Grindul Letea is mainly represented by grazing pastures and forests, containing 2,825 ha of a strictly protected area. The study was carried out from May to December (2013–2016). Ambient temperatures ranged from −13.4°C to 21.0°C (average of 6.2°C), with an average wind speed of 6 m/s and a maximum of 21 m/s. No immobilizations were carried out during heavy rain or snowfall. During heavy wind, the immobilizations took place in the leeward of the forest.

### Immobilization

Of the 220 darted horses, 91 horses (8 males, 83 females) were immobilized (lateral recumbency) after one dart injection. Remaining 129 horses were excluded due to darts bouncing or failure of drug injection. The body mass range was 80–375 kg based on body size and body condition (10) and the age range was 1–18 years based on dental wear. Horses involved in this study were grouped according to their estimated body weight (<200 kg, 200–300 kg, >300 kg). Seven females were assumed to be in late pregnancy and 10 females had a dependent foal. Males were immobilized either by mistake (*n* = 1), due to lack of sexual dimorphism (*n* = 5) or for a different medical intervention (*n* = 1). Three underaged horses (<2 years old, 2 females, 1 male) were darted unintentionally. Eighty-seven animals appeared healthy based on their behavior and physical appearance. Four females were underweight, with a poor body condition. More than 95% of the animals were members of established groups (harems).

Targeted animals were approached either by car or on foot and darted from 15 to 45 m using 6.0 ml aluminum darts (Pneudart Type “P”®, Pneudart, Inc., Wiliamsport, USA) and 3.81 cm wire barbed, 14 ga needles delivered by a 13-mm CO_2_-powered anesthetic rifle (X-Caliber® Gauged Projector, Pneudart, Inc. USA). Darts delivered in rump, neck or shoulder were considered as optimal hits, while those delivered in small muscle groups (abdomen, thorax, lower legs) or subcutaneously were considered as suboptimal hits. For the drug concentration in the aluminum darts, 1,000 mg of lyophilized ketamine (Ketamine 1 g, Kyron Laboratories, Johannesburg, South Africa) was dissolved into: a) 3 ml of 40 mg/ml® medetomidine (Kyron Laboratories) and b) 21 ml of 100 mg/ml ketamine (Vetased®, S.C. Pasteur, Filipesti de Padure, Romania or Ketamidor®, Richter Pharma AG, Wels, Austria). This 24-ml mixture contained 3,100 mg ketamine and 120 mg medetomidine contained a total volume of 24 ml and was divided into four 6.0-ml doses. The final concentration per 6-ml dart was 30 mg of medetomidine and 775 mg ketamine.

In 54 animals atipamezole (Antisedan® 5 mg/ml, Orion Corporation Animal Health, Turku, Finland) was administered intravenously for antagonism. Due to financial reasons, low doses of atipamezole (10–20 mg, 2–4 ml) were administered in 9 horses. Based on the observed effects, the dose of atipamezole was standardized to 25 mg (5 ml) per animal intravenously.

The following variables were recorded: induction time (time from darting to lateral recumbency); recumbency time (horse laying on its side) and recovery time (the time from administration of atipamezole until the horse was able to stand unassisted). First signs of induction occurred when ataxia and stilted gait were observed. Immobilized horses were blindfolded and positioned in lateral recumbency on flat ground, with the head and the front lower limb extended.

Anesthetic monitoring during immobilization included recording of rectal temperature, heart rate, respiratory rate, peripheral arterial hemoglobin saturation (SpO_2_) and capillary refill time (CRT) every 10 min until first recovery signs. SpO_2_ and heart rate were measured using a pulse oximeter (Nonin® 2500 A; Minneapolis, USA; or GIMA Oxy-100 VET®, Gessate, Italy) with the probe attached to the tongue. Respiration rate was recorded using a stethoscope or by observing the thoracic movements. Rectal temperature was recorded using a digital thermometer. Jugular blood and fecal samples were taken for pregnancy diagnosis. All mares were vaccinated with Porcine Zona Pellucida immunocontraceptive (ZonaStat-H®, Science and Conservation Center, Billings, MT, USA), ear tagged (Primaflex®, Caisley International GmbH, Bocholt, Germany) for later identification and age estimated based on dental wear (11). Samples for blood gases, acid-base status and hematology analyses were collected anaerobically from the auricular, facial, transverse-facial or hind-metatarsal arteries in 8 horses (7 females in weight group 200–300 kg, 1 male in weight group >300 kg). The samples were collected 15 and 30 min post-induction, using a self-filling pre-heparinized syringes (PICO™ 70, Radiometer Copenhagen, Brønshøj, Denmark) and analyzed with an I-STAT®1 Portable Clinical Analyzer (Abbott Laboratories, Abbott Park, Illinois, USA) using I-STAT® CG8+ cartridges. To maintain operating temperatures (16–30°C), the analyzer was stored in an insulated container. Arterial blood lactate from the same sample was measured with a Lactate Pro® (Arkray Factory Inc., KDK Corp., Japan). Measured blood parameters included pH, partial pressure of peripheral arterial oxygen hemoglobin saturation (PaO_2_), partial pressure of arterial carbon dioxide, sodium, potassium, calcium, and glucose, total carbon dioxide, bicarbonate, base excess, SpO_2_, hematocrit (Ht) and hemoglobin (Hb).

Based on published literature for domestic horses (12), hypoxemia was defined as a SpO_2_ saturation of 60–90%. Severe hypoxemia was defined as < 60%. Tachypnea was defined as >20 breaths per min and tachycardia as >45 beats per min. Hyperthermia was defined if the rectal temperature was >39°C.

### Data Analysis

Descriptive analyses (means, standard deviations, medians and range) were calculated. To assume Gaussian distribution, normality was checked by Shapiro-Wilks normality test. Depending on the distribution, one-way analysis of variance Kruskal-Wallis or ANOVA tests were performed to assess the medetomidine-ketamine protocol effect on physiologic parameters on body weight groups and age groups. To compare differences in medians between initial and final acid-base and hematologic parameters, a paired Wilcoxon rank sum test was used. A *p*-value of <0.05 was considered as statistically significant (95% confidence interval). To evaluate correlations between induction time, estimated body mass, age, recovery and recumbency time, a Kendall's or Spearman correlation test was performed, depending on the distribution. Statistical analysis was performed with the software R version 4.0.2 (13).

## Results

### All Horses

The mean induction time of all horses was 8.4 ± 5.4 min. The median time of lateral recumbency was 67 (range 35.0–112.0 min). Except the CRT with a mean value of 1.4 ± 0.6 s, all physiological parameters showed changes compared to values of anesthetized domestic horses (12). Physiological measurements, doses and times are grouped by body weight (Table 1).

**Table 1:** Doses, age, times and physiological measurements (given in mean ± SD or median and range) during anesthesia of feral horses in Romania, grouped by body weight.

**Variable**	***n***	** <200 kg**	***n***	**200–300 kg**	***n***	**>300 kg**
		**mean ± SD or median (range)**		**mean ± SD or median (range)**		**mean ± SD or median (range)**
Age (years)	5	1.5 (1.0–2.0)	70	5.4 ± 3.2	16	7.0 (3.0–12.0)
K (mg) i.v.	0	-	8	450.0 (400.0–800.0)	2	450.0 (400.0–500.0)
A (mg) i.v.	5	32.0 ± 9.8	41	25.0 ± 3.3	11	22.7 ± 4.7
IT (min)	5	3.6 ± 0.9	70	8.2 ± 4.5	16	10.6 ± 8.0
R (min)	5	88.0 (48.0–96.0)	70	65.8 ± 12.1	16	69.0 (54.0–80.0)
Recovery (min)	5	3.8 ± 3.6	39	3.6 ± 2.5	10	5.1 ± 5.8
HR (bpm)^a^	5	58.2 ± 4.1	67	47.3 ± 9.3	16	43 (30.0–63.0)
RR (breaths)^b^	5	26 (23.0–39.0)	68	26.5 ± 7.8	16	27 (16.0–40.0)
Temp (^0^C)^c^	5	37.7 (37.0–38.2)	66	37.8 (35.6–39.9)	15	37.2 (36.1–39.0)
SpO_2_ (%)^d^	5	86.0 (83.0–92.0)	61	88.0 (71.0–98.0)	14	86.5 (82.0–93.0)
CRT (sec)^e^	5	1.0 (1.0–1.0)	70	1.4 ± 0.7	15	1.23 ± 0.6

*K, Ketamine (additional dose intravenously); A, Atipamezole (total dose intravenously); IT, induction time; R, recumbency time; HR, heart rate; bpm, beats per minute; RR, respiratory rate; Temp, body temperature; SpO_2_, peripheral arterial oxygen hemoglobin saturation; CRT, capillary refill time; SD, standard deviation*.

a *beats per minute;*

b *breaths per minute;*

c *celsius;*

d *proportion of oxyhemoglobine;*

e *seconds*.

### Horses Where It Went Well

Seventy-nine dart injection sites (87%) were considered to be optimal, while twelve (13%) were considered suboptimal. There was no significant difference in the induction time between these groups. Eighty-one horses (98%) were completely immobilized with good muscle relaxation and with a mean induction time of 7.2 ± 4.2 min.

Animals with a lower estimated body mass had a shorter induction time (Spearman correlation test; *p* = 0.016). Spontaneous ear twitching and movement of the legs were observed occasionally in some horses at any time during recumbency. Ten horses required supplemental doses of ketamine due to incomplete immobilization or signs of spontaneous recovery 35–40 min post-induction.

Tachypnoea was recorded in 72 horses (79%). Nine animals (13%) showed apneustic patterns, characterized by intermittent period of apnea lasting 10–20 s, and respiratory stridor. While immobilized, 46 horses (51%) had tachycardia with regular rhythm. SpO_2_ values below 90% saturation were recorded in 56 animals (62%). Blood gas values are given in Table 2. In the second blood sample, blood lactate concentration increased beyond maximal limits (>1.5 mmol/L), while high glucose concentration (159 and 2 × 166 mmol/L) were registered in three horses. Bicarbonate, total carbon dioxide and base excess were higher in the second sample, whereas SpO_2_ and calcium values were lower. Low hematology parameters (Ht < 30%, Hb < 10 g/dl) were encountered in all horses, in both measured samples.

**Table 2:** Blood gases, acid base and hematologic parameters of eight feral horses, measured 15- and 30-min post-induction.

**Variable^**a**^**	***n***	**Sample 1**	***n***	**Sample 2**	***p*-value^**a**^**
		**mean ± SD or median (range)**		**mean ± SD or median (range)**	
pH	8	7.41 (7.37–7.50)	8	7.40 (7.33–7.49)	NSD
PaCO_2_ (mmHg)	8	43.1 (37.7–47.8)	8	47.0 (39.0–53.9)	NSD
PaO_2_ (mmHg)	8	53.0 (43.0–74.0)	8	42.5 (39.0–48.0)	0.01
BE	8	2.5 ± 2.8 (0.0–8.0)	8	4.5 (1.0–10.0)	0.03
HCO_3_ (mmol/L)	8	27.5 (25–31.9)	8	29.6 (26.1–34.0)	0.02
TCO_2_ (mmol/L)	8	29.0 (26.0–33.0)	8	31.0 (27.0–35.0)	0.04
SpO_2_ (%)	8	87.0 (80.0–94.0)	8	77.0 (73.0–85.0)	0.01
Lac (mmol/L)	7	2.9 ± 2.2	8	3.2 (1.6–6.6)	NSD
Na (mmol/L)	8	131.5 (128.0–135.0)	8	132.0 ± 1.8	NSD
K (mmol/L)	8	4.5 ± 0.3	8	4.6 (3.6–5.0)	NSD
iCa (mmol/L)	8	1.4 (1.3–1.6)	8	1.4 (1.3–1.5)	0.03
Glu (mg/dl)	8	116.0 (89.0–166.0)	8	139.0 (89.0–186.0)	0.02
Hct (%)	8	26.1 ± 5.1	8	27.0 (23.0–30.0)	NSD
Hb (g/dl)	8	8.9 ± 1.7	8	9.2 (7.8–10.2)	NSD

a *NSD, no significant difference (paired Wilcoxon signed-rank-test); PaCO_2_, partial pressure of arterial carbon dioxide; PaO_2_, partial pressure of arterial oxygen, BE, base excess; HCO_3_, bicarbonate; TCO_2_, total carbon dioxide; SpO_2_, peripheral arterial hemoglobin oxygen saturation; Lac, lactate; Na, blood concentrations of sodium; K, potassium; iCa, ionized calcium; Glu, glucose (normal range 62–134 mmol/L); Hct, hematocrit; Hb, hemoglobin*.

Fifty-eight immobilizations were antagonized with 25 mg atipamezole per animal intravenously after approximately $\bar{\text{X}}$ = 60.5 ± 12.8 min of anesthesia. Following the administration, the horses stood within $\bar{\text{X}}$ = 3.9 ± 3.4 min (*n* = 54). Most horses had a smooth recovery, with one (*n* = 86; 95%) or more (*n* = 5; 5%) attempts at standing. The transition to sternal recumbency in many cases was assisted, by positioning the horses on the sternum with both front limbs extended. Once in standing position, the animals preferred to remain stationary and moving atactic when stimulated. Most recoveries were uneventful (*n* = 89; 98%). There was no significant differences in induction, recumbency or recovery times between the body mass groups.

### Outliers

The three underaged horses achieved lateral recumbency within 3–4 min.

Five horses (5%) showed prolonged induction times with periods of incoordination before immobilization and lateral recumbency. Two of them had to be blindfolded before they became recumbent.

One case presented alkalemia (pH = 7.50) that was observed in both samples. Body temperature increased (>39°C) in 6 individuals (7%), while one case of severe hypoxemia was associated with shallow respiration. This mare was positioned on the sternum, atipamezole was administered intravenously, and respiration was stimulated by acupuncture (prime vessel 26). The mare remained in sternal position during the entire procedure with significant improvement in respiration pattern and SpO_2_.

One male showed signs of lameness and paresis of the left front leg but recovered after 1 h. One mare was euthanized the day after immobilization due to quadriparesis of unknown etiology, likely caused by capture myopathy. The animal was not able to stand up after the immobilization despite a total dose of 75 mg atipamezole and of several efforts of suspending it in slings. An additional dose of atipamezole was given between 41 and 64 min after the initial dose in 3 (3%) horses having prolonged recoveries.

## Discussion

The anesthetic protocol chosen in the present study demonstrated a reliable immobilization in free-ranging horses by good muscle relaxation and a mean induction time of 8.4 min. After a mean recumbency time of 67 min, 98% of the recoveries were uneventful. With the exception of CRT, all physiological parameters showed changes compared to values of anesthetized domestic horses (12). The most concerning physiological change was SpO_2_ values below 90% saturation in 62% of the animals.

### Drugs and Dose

The doses of medetomidine and Ketamine were selected based on the authors' previous experience with this drug combination in free-ranging feral horses. The combination demonstrated a reliable immobilization, with a good anesthesia plane in the majority of cases, while offering broad safety margins. These findings support published reports of medetomidine-ketamine combinations in other equids (4, 6, 8) and are reinforced by the three underaged horses with lower body weight, that were immobilized unintentionally with 7.4 mg/kg ketamine and 0.3 mg/kg medetomidine), achieving lateral recumbency in $\bar{\text{X}}$ = 3.3 min, and remaining recumbent for $\bar{\text{X}}$ = 92.6 min while recovering uneventfully.

### Induction Time and Lateral Recumbency

We did not find any significant difference between optimal and suboptimal injection sites contrary to studies which showed that one of the most important determinants for quick induction is dart placement causing better absorption of the drugs (14–16). Individual animal responses on anesthetic drugs can be based on their condition and appear to have a more significant impact on induction times (3). This could explain the correlation of body mass and induction time with the need for additional ketamine in some horses. In these cases, the animals showed signs of anesthesia, but managed to remain standing for a longer period, presumably wearing off the effect and duration of anesthesia. Once recumbent, clinical signs, like ear twitching, leg and tail movements and responses to external stimuli, were considered signs of early recovery, requiring additional ketamine intravenously.

The mean induction time was 10.6–0.6 min shorter than reported in other studies which is most likely caused by the different approach methods e.g., helicopter pursuit, species and doses (4, 6–8).

A lack of animal response, muscle relaxation, jaw muscle tone decrease, and positive anal reflex indicated a proper depth of anesthesia, which facilitated blood sampling, ear tagging, micro chipping and immunocontraceptive administration. Furthermore, this protocol might enable short surgical procedures.

### Physiological Changes During Maintenance

Potent alpha-2 adrenoceptor agonists are known to cause some degree of bradycardia and bradypnea, but the immobilization of free-ranging horses with medetomidine-ketamine resulted in hypoxemia with tachycardia and tachypnea for more than a half of subjects, probably due to cardioacceleratory properties of ketamine (6). Despite tachypnea, hypoxemia indicated inadequate ventilation and gas exchange. Movement of the chest wall denoted that gas was inhaled, but the volume of gas exchange could have been low (12). The reduction of lung volume and asynchronous ventilation were also related to the lateral posture during general anesthesia (17) which especially applies to pregnant mares, as the distended abdominal content causes noticeable ventilation problems. Respiratory insufficiency is a known side effect in equine anesthesia (18) but supplemental oxygen may not be feasible under field conditions (5). However, in respect of severe consequences especially for potential anemic or pregnant animals, we recommend portable oxygen containers. The lowest possible oxygen flow needed to maintain normoxia should be used with values below maximal fraction of inspired oxygen (FIO_2_ = 1) to avoid absorption atelectasis (18, 19).

Apneustic pattern of breathing was observed in nine horses associated with respiratory stridor. While an irregular breathing pattern is a consequence of ketamine anesthesia, the respiratory stridor occurred because of upper airway muscle relaxation, causing partial occlusion of the airways (20). In clinically normal horses, medetomidine can inhibit the central respiratory system in anesthetized and recumbent horses (21). In this study, evidence of central respiratory inhibition was also correlated both with a severe drop of arterial PO_2_ and hypercapnia along with increases of bicarbonate, total carbon dioxide, and base excess. Based on these results of eight horses, seven were diagnosed with acute respiratory acidosis (12).

Elevated body temperature in anesthetized horses is considered a rare, but severe, phenomenon (22). Hyperthermia was seen in 6 of 91 horses, the most likely cause being a stress-related increased metabolic rate due to capture, pursuit and anesthesia. This hypothesis is supported by increased blood glucose levels, as seen in three horses. However, the rectal temperatures of the animals remained within the published normal range (23).

The inadequate state of tissue oxygenation could explain the progressive increase of blood lactate (24), seen in 7 of 8 horses.

Ht and Hb in all sampled horses were below the physiological range (Ht < 30%, Hb < 10 mg/dl), suggesting anemia. Anemia can be correlated with the presence of equine infectious anemia (EIA), a disease caused by a non-oncogenic retrovirus transmitted by hematophagous parasites. In an independent blood test on 55 captured feral horses in 2011, 20% were positive for EIA (Rosu, unpublished data).

On the other hand, we have not found any published data regarding normal hematology values in Danube Delta horse population, the low Ht and Hb values found in all horses might not necessarily suggest an underlying pathology; these values could be within the normal range in this population.

The case with signs of exertional myopathy could be caused by a ventilation-perfusion mismatch associated with an altered tissue blood flow with cessation in aerobic metabolism, secondary to hypotension and low cardiac output (12).

### Recovery and Antagonization

Medetomidine was antagonized with atipamezole in 54/91 horses. To keep overall immobilization costs down, the effective medetomidine:atipamezole ratio used (1:1.2) was lower than reported in other studies for equids [*Equus caballus domesticus*; 1:5 to 1:10 (25); *Equus ferus asinus*, 1:2.5 (8); *Equus przewalskii*; 1:2 (7); *Equus ferus caballus;* 1:2; (4)] or other wild animal species [1:5; (6)]. However, the dose of 25 mg atipamezole per animal gave an acceptable recovery and supported the suggestion that recommended doses for domestic horses tend to be larger than clinically necessary (25). Most recoveries were uneventful, with one or more attempts for standing, however, the horses presented prolonged ataxia, remaining stationary for varied periods. The timing and the route of antagonist administration were chosen based on previous reports (3, 6, 8).

### Limitations

One limitation of this study was the absence of blood pressure measurements, which we recommend for future studies. Low arterial blood pressures (< 60 mm Hg) can be caused by the use of anesthetics and are associated with an increased potential of complications like rhabdomyolysis.

Furthermore, the results might be gender biased as the majority of horses were females. An increased sample size of blood gas values would have improved the statistical power for our analyses, but it was not considered due to financial reasons.

### Conclusions

We have concluded that the given drug mixture of 30 mg medetomidine and 775 mg ketamine per animal provides an adequate anesthesia for 67 min. In addition, a lower dose of atipamezole is sufficient for antagonism. The systemic compromises produced by deficient ventilation during recumbency are well-tolerated in healthy animals. However, the observed severe hypoxemia and hypercapnia emphasized a potential risk in field anesthesia, especially for anemic animals. Thus, we recommend supplemental oxygen during field immobilization. In addition, horses should be positioned sternally if severe hypoxemia occurs (18).

## Data Availability Statement

The raw data supporting the conclusions of this article will be made available by the authors, without undue reservation.

## Ethics Statement

The animal study was reviewed and approved by National Sanitary Veterinary and Food Safety Authority (ANSVSA) under the research permit nr. 14924 from 15.10.2012 and the Danube Delta Biosphere Reserve Authority (ARBDD) collaboration protocol nr. 160/26.05.2015.

## Author Contributions

OR, JA, AE, and SK initiated the study and designed the experiments. OR, AE, SK, and IM contributed during fieldwork and data collection. Equipment was provided by JA and OR. Data management was done by OR, IM, AE, and SK. Data analyses and preparation of was done primarily by SK, with contributions from IM. OR, IM, and SK drafted the manuscript. AE and JA critically revised the manuscript. All authors participated in revisions and approved the final manuscript.

## Conflict of Interest

The authors declare that the research was conducted in the absence of any commercial or financial relationships that could be construed as a potential conflict of interest.

## Abbreviations

CRT: capillary refill time
FIO_2_: fraction of inspired oxygen
Hb: hemoglobin
Ht: hematocrit
IT: induction time
PaCO_2_: partial pressure of peripheral carbon dioxide
PaO_2_: partial pressure of peripheral arterial oxygen saturation
SpO_2_: peripheral arterial oxygen hemoglobin saturation.
